# Research advances on estrogen deficiency leading to depression

**DOI:** 10.1515/tnsci-2025-0399

**Published:** 2026-05-13

**Authors:** Chunjiao Wang, Yanfen Chang, Hui Sheng

**Affiliations:** College of Basic Medicine, Naval Medical University, Shanghai, China; Department of Physiology, College of Basic Medicine, Naval Medical University, Shanghai, China

**Keywords:** depression, estrogen, gut-brain axis, HPA axis, neuroinflammation

## Abstract

Depression is a debilitating mental disorder with a significantly higher prevalence in women, particularly during periods of hormonal fluctuation such as perimenopause, postpartum, and postmenopause. Estrogen, especially 17β-estradiol (E_2_), serves as a crucial neuroactive steroid that regulates mood, cognition, and neural homeostasis through nuclear and membrane-associated receptors. Accumulating evidence suggests that estrogen deficiency contributes to the pathogenesis of depression via multiple interconnected mechanisms, including dysregulation of monoamine neurotransmission, hyperactivation of the hypothalamic–pituitary–adrenal (HPA) axis, decreased brain-derived neurotrophic factor (BDNF) expression, impaired mitochondrial function and bioenergetics, neuroinflammation mediated by glial cells, and disruption of gut–brain axis communication. These alterations collectively lead to synaptic dysfunction, reduced neuroplasticity, and increased neuronal vulnerability. Therapeutic strategies such as estrogen replacement therapy (ERT), selective estrogen receptor modulators (SERMs), and receptor-specific agonists show promising antidepressant effects, particularly when administered during critical windows of hypoestrogenism. This review systematically elaborates the pathophysiological mechanisms underlying estrogen deficiency-induced depression and discusses recent advances in estrogen-based therapeutics, highlighting future directions for targeted and personalized treatment approaches.

## Introduction

**Figure 1:**
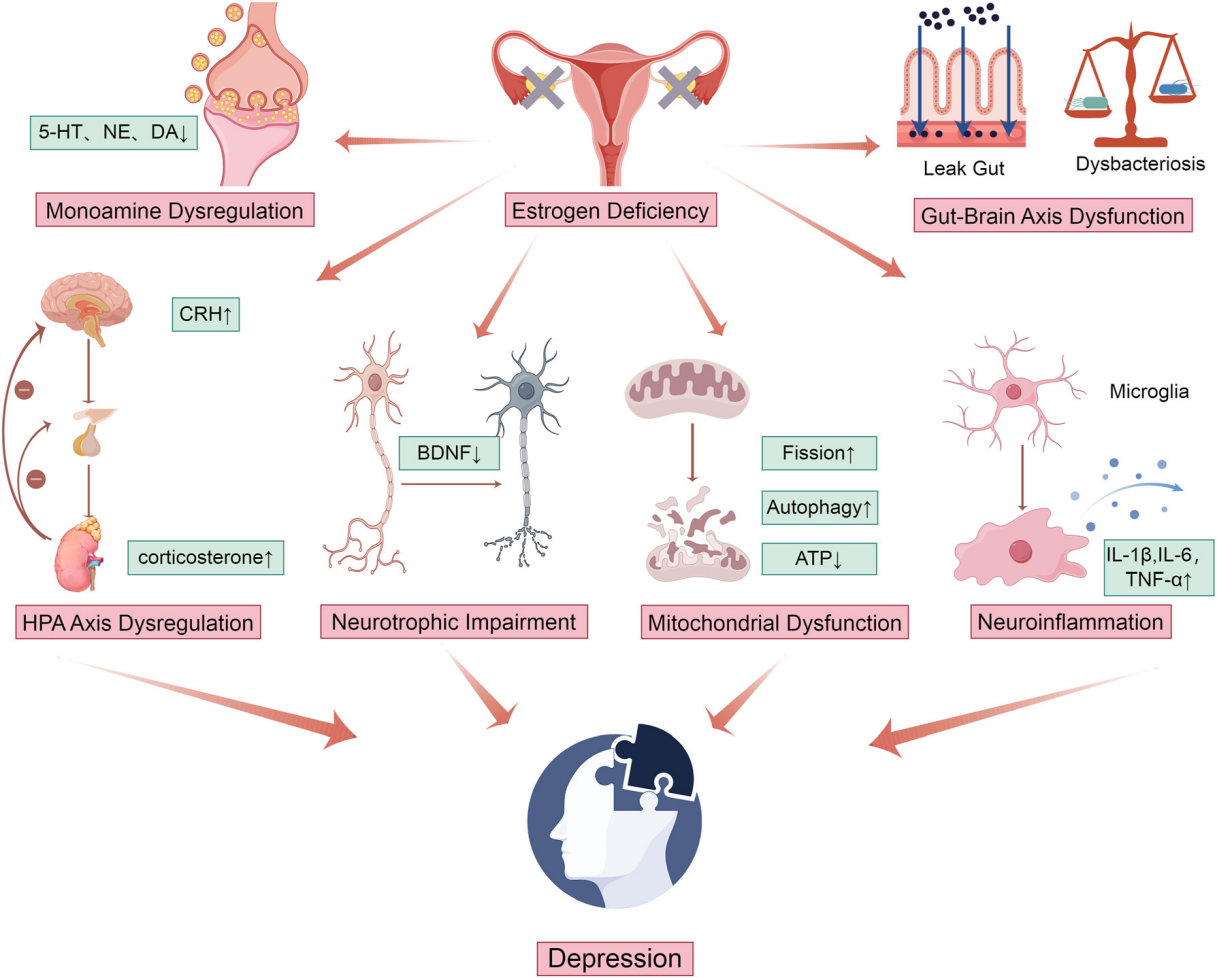
Integrated pathophysiological mechanisms of estrogen deficiency-induced depression.

Depression is a prevalent mood disorder affecting approximately 4 % of the global population, characterized by persistent low mood, anhedonia, cognitive impairment, and suicidal ideation. As a leading cause of disability worldwide, its economic burden is projected to double by 2030 [1]. Women bear a disproportionate burden, with epidemiological studies indicating a higher lifetime prevalence compared to men, a disparity that fluctuates with reproductive stages [2], 3]. Notably, the risk of depression is significantly elevated during periods of estrogen fluctuation or decline, such as the premenstrual, postpartum, and perimenopausal phases [4], [5], [6], [7], [8], suggesting a pivotal role for this hormone in mood regulation (Figure 1).

Estrogen, particularly E_2_, is a steroid hormone that functions as a key neuroregulator. It mediates its effects through classical nuclear receptors, estrogen receptor α (ERα) and estrogen receptor β (ERβ), and the membrane-associated G protein-coupled estrogen receptor (GPER). ERα and ERβ are differentially distributed in the brain: ERα is abundant in the hypothalamus, while ERβ is highly expressed in the hippocampus and cortex – regions critical for mood and cognition. Through these receptors, estrogen influences neuroprotection, synaptic plasticity, and mood [9]. Substantial evidence indicates that declining or fluctuating estrogen levels impairs cognitive function and emotional processing, thereby increasing susceptibility to depression [10], [11], [12], [13].

In recent years, the link between estrogen deficiency and depression has garnered significant research interest. While numerous studies have investigated this association, the underlying mechanistic pathways remain to be fully elucidated. Several excellent reviews have summarized the role of estrogen in depression; however, the present review offers a distinct integrative perspective. We focus on the intricate crosstalk between traditional pathways (monoamine, HPA axis, BDNF) and emerging mechanisms, particularly mitochondrial dysfunction and gut-brain axis dysregulation, in the context of estrogen deficiency. By systematically linking these interconnected mechanisms to current and future therapeutic strategies, this review provides a comprehensive framework for understanding and targeting estrogen deficiency-induced depression.

## Mechanisms of depression induced by estrogen deficiency

### Dysregulation of the monoamine neurotransmitter systems

The monoamine hypothesis, which posits that depression arises from deficient neurotransmission of serotonin (5-HT), norepinephrine (NE), and dopamine (DA), remains a cornerstone of depression research. It is widely recognized that estrogen is a potent modulator of these systems, through which estrogen regulates the emotions.

Research has shown that estrogen profoundly influences serotonergic signaling. Clinical studies using magnetic resonance spectroscopy (MRS) further demonstrated that estrogen-progestogen therapy activates 5-HT pathways in some brain regions, correlating with improved mood and cognition [14]. Moses-Kolko et al. also reported that hormone therapy (estradiol combined with micronized progesterone) increased 5-HT_2A_ receptor density in postmenopausal women [15]. Wang et al. observed a widespread reduction in 5-HT expression across brain regions in ovariectomized (OVX) mice [16], a finding corroborated in the prefrontal cortex and hypothalamus of OVX mice subjected to chronic stress by Zhang et al. [17]. At the molecular level, Gu et al. found that estrogen deficiency upregulates the plasma membrane monoamine transporter (PMAT) via the Mitogen-Activated Protein Kinase/Extracellular signal-Regulated Kinase (MAPK/ERK) pathway, increasing 5-HT reuptake in hippocampus, an effect reversed by estrogen supplementation [18]. Furthermore, estrogen enhances the synthesis of 5-HT by regulating the rate-limiting enzyme tryptophan hydroxylase (TPH) [19], 20]. Estrogen also supports serotonergic neurons by upregulating brain-derived neurotrophic factor (BDNF) [21], [22], [23]. Its deficiency thus leads to weakened serotonergic tone, manifesting as core depressive symptoms.

The noradrenergic and dopaminergic systems are also estrogen-sensitive. Studies indicate that estrogen elevates NE and DA levels by upregulating synthetic enzymes like tyrosine hydroxylase (TH) and dopamine β-hydroxylase (DBH), while inhibiting metabolic degradation [24], [25], [26], [27], [28]. For instance, in rodent models, OVX inhibits DBH and NE in the ventral hippocampus, cortex, and hypothalamus, an effect that is reversible with E_2_ treatment [29], [30], [31]. Furthermore, in OVX monkeys, Pau et al. reported that E_2_ administration increased TH mRNA in the locus coeruleus and NE release in the hypothalamus [32]. Additional preclinical evidence indicates that estrogen modulates dopamine transporter (DAT) and receptor sensitivity [33]. Liu et al. observed reduced lateral habenula DA concentrations and increased depressive-like behaviors in OVX rats, which were restored by E_2_ treatment [34].

Consequently, estrogen functions as an efficient “regulator”, meticulously maintaining the equilibrium and functionality of monoamine neurotransmitter systems. Estrogen deficiency leads to weakened serotonergic, noradrenergic, and dopaminergic tones, manifesting as core depressive symptoms such as anhedonia, fatigue, impaired concentration, and psychomotor retardation. Importantly, the decline in monoamine signaling does not occur in isolation; it can further impair mitochondrial function and exacerbate neuroinflammation, creating a vicious cycle that amplifies neuronal vulnerability and contributes to the persistence of depressive symptoms [35].

### Dysregulation of the HPA axis

Hyperactivity of the HPA axis is a well-established feature of depression [36], 37]. Recent studies have confirmed that this dysregulation is also present in postmenopausal and postpartum depression [7], 38], 39]. Estrogen typically exerts an inhibitory effect on the HPA axis by enhancing glucocorticoid receptor (GR) negative feedback sensitivity and suppressing corticotropin-releasing hormone (CRH) expression.

Human and animal studies provide direct evidence for this regulation of estrogen in depressive models. A clinical study noted an increased incidence of depression in women using oral contraceptives, potentially linked to suppressed estradiol and subsequent HPA axis dysregulation [40]. Ma et al. observed HPA axis hyperactivation in an OVX-chronic stress rat model [41]. In this regard, Cleber et al. demonstrated that estrogen treatment decreased CRH mRNA expression in OVX rats, suppressing HPA axis activity [42]. Consistent with this, Daodee et al. and Eid et al. reported elevated corticosterone in OVX mice and its reduction after estradiol treatment, respectively [43], 44]. Noteworthy, Wang et al., using a simulated postpartum depression model, found reduced hippocampal GR expression alongside depressive behaviors [45].

These findings collectively suggest that estrogen deficiency leads to HPA axis hyperactivation, resulting in elevated cortisol/corticosterone levels and impaired negative feedback. This chronic stress-like state contributes to the development of depressive symptoms, particularly those related to stress sensitivity and mood instability. Furthermore, HPA axis hyperactivity synergizes with neuroinflammation and gut-brain axis dysregulation to perpetuate a state of chronic stress and systemic immune activation, with elevated glucocorticoids promoting pro-inflammatory responses and increased intestinal permeability, thereby establishing a self-reinforcing loop that sustains both peripheral and central pathology [46].

### Neurotrophic deficits and neuronal impairment

Decades of research have documented increased cellular dysfunction and impaired neuroplasticity in the cerebral cortex and limbic systems of individuals with depression [47], [48], [49]. BDNF is crucial for neuronal survival and plasticity, and its reduction is extensively documented in depression [50], [51], [52]. Prior studies have strongly suggested that estrogen’s core actions involve the regulation of neurotrophic factors and the maintenance of neuroplasticity.

Estrogen is a key regulator of BDNF, as evidenced by the correlation between declining estrogen levels and reduced BDNF concentrations [23], 53], 54]. Experimentally, Baek et al. observed significantly suppressed BDNF expression in the hippocampus of OVX mice [55], while other studies confirmed that E_2_ supplementation increases BDNF in the prefrontal cortex and hippocampus, alleviating depressive-like behaviors [56], [57], [58]. Of note, some mechanistic studies showed that estrogen can induce BDNF expression by binding directly to estrogen response elements (EREs) on the BDNF gene [59], 60]. Furthermore, Deb et al. demonstrated that E_2_ activates multiple BDNF promoters via histone acetylation, and then modulates hippocampal function [61], 62].

Beyond BDNF, estrogen directly influences neuronal health and autophagy. Research indicates that OVX induces altered neuroplasticity and weakened synaptic transmission [63]. Pandey et al. found enhanced hippocampal autophagy in OVX rats, which was mitigated by estradiol treatment, restoring cognitive function [64]. Other studies suggest that estrogen can suppress detrimental autophagy and protect against oxidative stress and neuronal degeneration [65], [66], [67].

Thus, estrogen deficiency contributes to decreased BDNF expression and impairs key neuroprotective processes such as autophagy regulation. These deficits compromise synaptic plasticity and neuronal resilience, forming a cellular basis for depressive symptomatology. Given that BDNF signaling is exquisitely sensitive to both inflammatory cytokines and mitochondrial bioenergetic status, its deficiency represents a critical point of convergence where multiple upstream pathways – including HPA axis hyperactivity, neuroinflammation, and mitochondrial dysfunction – jointly impair neuronal resilience and amplify the risk of depressive pathology [68].

### Mitochondrial dysfunction

Mitochondrial dysfunction is closely associated with depression. Research has revealed that estrogen receptors are present in brain mitochondria, and that estrogen is vital for maintaining mitochondrial health [69], 70]. Recently, emerging evidence has indicated that estrogen loss is involved in mitochondrial structural and function abnormalities [71]. Indeed, estrogen can regulate mitochondrial structure and function through multiple pathways.

Estrogen plays a critical role in the regulation of mitochondrial dynamics and quality control. Ovariectomy and aging exacerbate mitochondrial fragmentation and promote the formation of abnormal ring-shaped mitochondria in the prefrontal cortical synapses of monkeys, correlating with working memory deficits, which are reversible with E_2_ treatment, underscoring estrogen’s essential role in maintaining mitochondrial dynamics [72]. Studies have shown that both ERα and ERβ are detectable in brain mitochondria, with ERβ being predominant and implicated in intricate regulatory functions [73], 74]. For instance, it has been shown that ERβ interacts directly with A-kinase-anchoring protein 1 (AKAP1), activating a PKA-mediated signaling cascade that enhances oxidative phosphorylation and suppresses fission [75], 76]. Consistent with this, E_2_-induced overexpression of ERβ in T47D cells elevated oxidative phosphorylation complex proteins and reduced fission [77]. Additionally, mitochondrial autophagy, essential for quality control and mediated largely by the PINK1/Parkin pathway, is also estrogen-sensitive. ERα stabilizes Parkin via HTRA2, preserving mitophagy [78]. Reduced PINK1 and Parkin in OVX rats imply impaired mitophagosome formation [79], while GPER1 overexpression elevates Parkin expression and enhances mitophagy [80]. Although some studies report increased mitophagy in OVX or ERα-silenced hippocampal neurons [64], the prevailing consensus confirms that estrogen deficiency ultimately disrupts mitochondrial autophagy, contributing to neuronal dysfunction.

Beyond dynamics, estrogen significantly influences mitochondrial respiratory function [81]. Proteomic analyses in female rats show that E_2_ modulates the expression of pyruvate dehydrogenase (PDH), oxidative phosphorylation complexes, and ATP synthase [82], [83], [84]. Experimental studies in OVX models demonstrate that E_2_ supplementation enhances mitochondrial respiration, partly mediated through Nrf2 activation, by upregulating cytochrome c and complex IV expression [85]. Furthermore, OVX is also associated with reduced expression of ERα and SIRT1, along with diminished ATP levels – all of which are restored following E_2_ treatment, demonstrating its protective role in preserving mitochondrial functional capacity [86], [87], [88].

In summary, estrogen deficiency triggers mitochondrial fragmentation, impairs oxidative phosphorylation, and disrupts mitophagy, leading to energy deficit and increased oxidative stress. These mitochondrial abnormalities compromise neuronal function and contribute to the pathophysiology of depression. Crucially, mitochondrial dysfunction does not occur in isolation; it both fuels neuroinflammation by releasing damage-associated molecular patterns (DAMPs) and is exacerbated by inflammatory cytokines, establishing a bidirectional loop that drives disease progression [89], 90]. This mutual reinforcement between mitochondrial impairment and neuroinflammation further amplifies synaptic dysfunction and neuronal vulnerability in the context of estrogen deficiency.

### Neuroinflammation and glial cell activation

Chronic neuroinflammation is increasingly recognized as a key component of depression. Studies indicate that estrogen has potent anti-inflammatory properties. The menopausal transition is accompanied by an elevation in systemic inflammation [91], 92]. It has been observed that menopausal women exhibit elevated systemic levels of pro-inflammatory cytokines (IL-1β, IL-6, TNF-α), which are reduced by hormone therapy [93], [94], [95], [96].

At the cellular level, Sanchez et al. suggested that estrogen inhibits microglial immune responses, thereby reducing susceptibility to adverse behavioral alterations; its deficiency induces neuroimmune activation [97]. Animal studies indicate that low E_2_ levels activate the Nuclear factor κB (NF-κB) pathway in microglia, promoting a pro-inflammatory M1 phenotype and the release of inflammatory cytokines [91], 98]. Jiang et al. discovered that in aged female mice challenged with lipopolysaccharide (LPS), reduced E_2_ downregulated the sirtuin 1 (SIRT1)/NF-κB pathway, and that E_2_ pretreatment or the ERα agonist administration activated this pathway to alleviate depressive behavior [99]. Similarly, it has been shown that estrogen treatment in early-middle-aged OVX rats suppressed NF-κB expression in the prefrontal cortex and striatum [100], 101]. It is worth noting that the anti-inflammatory effects are receptor-mediated: ERβ agonists modulate IL-6 and IL-1β levels and inhibit glial inflammation [102], [103], [104], ERα agonists suppress microglial activation and exert neuroprotective effects [105], [106], [107], and the GPER agonist G1 reduces pro-inflammatory cytokines in microglia [108], 109].

Collectively, these findings suggest that estrogen deficiency exacerbates neuroinflammation by promoting microglial pro-inflammatory polarization and cytokine release. This neuroinflammatory milieu can directly impair neuronal function and synaptic plasticity, contributing to depressive pathogenesis. Moreover, this neuroinflammatory milieu does not merely result from estrogen deficiency but actively contributes to other pathological processes: it impairs monoamine synthesis by disrupting tryptophan metabolism, disrupts mitochondrial function through oxidative stress, and compromises BDNF signaling, further underscoring the interconnected and self-reinforcing nature of these mechanisms [110].

### Dysregulation of the gut-brain axis

The gut-brain axis represents a critical communication network, whose core function lies in the maintenance of equilibrium by the gut microbiota, intestinal barrier, and immune system. Recent studies have revealed its dysregulation inhibits serotonin synthesis, disrupts HPA axis function, and impairs neural plasticity, ultimately leading to depressive mood and behavior [111], [112], [113]. Interestingly, estrogen has been implicated to play a crucial role in maintaining the gut-brain axis homeostasis.

Researchers have found that gut permeability fluctuates with estrogen levels during the estrous cycle in rats [114], [115], [116]. Wu et al. and others have reported that estrogen levels correlate with microbial diversity in postmenopausal women [117], 118]. Studies in OVX mice have shown that estrogen deficiency alters the gut microbiota, increasing the Firmicutes/Bacteroidetes ratio and the abundance of pro-inflammatory species, while estrogen supplementation promotes beneficial bacteria like Bifidobacteria and Akkermansia [119], [120], [121], [122]. Guan et al. observed that this dysbiosis leads to increased LPS levels [123], 124]. Pretorius et al. demonstrated that estrogen can suppress gut inflammation by inhibiting microbial trace amine production [125]. The ensuing “leaky gut” and systemic inflammation can trigger neuroinflammation and central nervous system dysfunction, thereby contributing to depression.

Therefore, estrogen deficiency-induced gut dysbiosis and increased intestinal permeability establish a pro-inflammatory state that communicates with the brain via the gut-brain axis. This pathway represents a novel mechanistic link between peripheral and central pathophysiology in depression. Collectively, gut-brain axis dysregulation serves as a critical upstream trigger that links peripheral inflammation to central neuroinflammatory, neuroendocrine, and metabolic disturbances [126]. By integrating signals from the external environment (e.g., diet, stress) with internal immune and endocrine responses, it functions as a key hub that orchestrates multiple downstream pathological mechanisms, including HPA axis hyperactivity, neuroinflammation, and mitochondrial dysfunction, ultimately contributing to the depressive phenotype.

## From mechanisms to treatment: therapeutic strategies

The multifaceted pathophysiological mechanisms linking estrogen deficiency to depression provide a strong rationale for estrogen-based therapeutic interventions. Estrogen replacement therapy (ERT) remains the most direct approach. Numerous randomized controlled trials demonstrate that transdermal E_2_ is effective in alleviating depressive symptoms in perimenopausal and postpartum women, particularly those with vasomotor symptoms [127], [128], [129]. These findings support the “therapeutic window” hypothesis, which suggests maximal benefit when treatment is initiated early in the hypoestrogenic period [130], [131], [132]. This temporal dependency is robustly supported by both preclinical and clinical data, underscoring the importance of timely intervention.

The clinical application of ERT, however, requires a nuanced understanding of several key factors that influence its therapeutic outcomes and risk profile. First, the route of administration matters considerably: transdermal estradiol avoids first-pass hepatic metabolism, resulting in more stable serum hormone levels and a lower risk of thromboembolism compared to oral formulations [128]. This more physiological hormone profile makes transdermal delivery particularly advantageous for managing mood disorders. Second, the risk-benefit ratio must be carefully weighed in clinical decision-making. While ERT carries well-documented risks – such as increased breast cancer risk with prolonged use and venous thromboembolism, especially in older women – it is equally essential to consider the substantial risks associated with untreated depression. These include cardiovascular morbidity, cognitive decline, impaired quality of life, and elevated suicide risk [133]. Notably, updated analyses from large-scale trials, such as the Women’s Health Initiative, suggest that for recently menopausal women, the benefits of ERT may outweigh the risks for many individuals. Third, treatment must be personalized based on patient age, symptom profile, medical history, and personal preferences. For women with a uterus, estrogen must be combined with a progestogen to prevent endometrial hyperplasia, a regimen that may introduce additional mood-related side effects requiring careful monitoring.

However, ERT’s long-term safety concerns limit its use [133]. To overcome these limitations, research has shifted towards targeted strategies. Among these, selective ERβ agonists have emerged as particularly promising candidates. ERβ activation promotes neurogenesis and exerts anti-inflammatory effects without the proliferative risks of ERα activation. Preclinical studies have demonstrated that ERβ-selective compounds effectively ameliorate depressive-like behaviors in ovariectomized rodent models, supporting their potential as novel antidepressants [134], [135], [136], [137], [138]. Phytoestrogens and SERMs like raloxifene offer alternative pathways with tissue-selective activities, though their antidepressant efficacy requires further validation in large-scale trials [139], [140], [141], [142], [143], [144]. Preliminary studies suggest potential antidepressant properties of SERMs, but further research is needed to establish their clinical utility in treating estrogen deficiency-associated depression.

Collectively, these emerging therapeutic avenues, grounded in the mechanistic insights discussed in preceding sections, hold promise for more targeted and safer interventions. As summarized in Table 1, each pathophysiological mechanism presents potential therapeutic targets, and the development of receptor-specific and tissue-selective compounds represents a logical progression toward personalized medicine in this field.

**Table 1:** Summary of key pathophysiological mechanisms and corresponding therapeutic strategies in estrogen deficiency-induced depression.

Mechanism	Key pathological changes due to estrogen deficiency	Corresponding therapeutic strategies/targets
Monoamine dysregulation	↓ Synthesis of 5-HT, NE, DA ↑ Reuptake of 5-HT ↓ 5-HT receptor density ↓ Neurotrophic support to monoamine neurons	– Estrogen Replacement Therapy (ERT) – Selective Estrogen Receptor Modulators (SERMs) – ERβ-selective agonists (to modulate TPH2/5-HT pathway)
HPA axis hyperactivity	↓ Glucocorticoid receptor (GR) sensitivity (impaired feedback) ↑ CRH expression ↑ Corticosterone/Cortisol levels	– ERT (to restore GR function and suppress CRH) – GR-targeting agents
Neurotrophic deficits	↓ BDNF expression (via loss of direct ERE binding and epigenetic regulation) Impaired neuroplasticity and synaptic transmission Dysregulated autophagy (often ↑ detrimental autophagy)	– ERT (restores BDNF via ERα/ERβ) – ERβ agonists (promote neurogenesis) – Compounds activating CREB/BDNF pathway (e.g., some SERMs)
Mitochondrial dysfunction	↑ Mitochondrial fragmentation (abnormal ring-shaped mitochondria) ↓ Oxidative phosphorylation Impaired mitophagy (dysregulated PINK1/Parkin pathway) ↑ Oxidative stress	– ERT (improves bioenergetics via ERβ-AKAP1-PKA signaling) – ERβ-selective agonists (promote OXPHOS, reduce fission) – GPER1 agonists (enhance mitophagy)
Neuroinflammation & glial activation	↑ Microglial activation (M1 pro-inflammatory phenotype) ↑ Pro-inflammatory cytokines (IL-1β, IL-6, TNF-α) ↓ SIRT1, activation of NF-κB pathway	– ERT (suppresses NF-κB) – ERα agonists (suppress microglial activation)– ERβ agonists (modulate IL-6, IL-1β) – GPER1 agonist (G1) (reduces cytokines)
Gut-brain axis dysregulation	Altered gut microbiota (↑ Firmicutes/Bacteroidetes ratio, ↑ pro-inflammatory species, ↓ beneficial bacteria like Akkermansia) ↑ Intestinal permeability (“leaky gut”) ↑ Systemic LPS and inflammation	– ERT (promotes beneficial bacteria) – Probiotics / Prebiotics – Dietary interventions (e.g., phytoestrogens)
Direct therapeutic interventions	Integration of all above mechanisms	– Estrogen Replacement Therapy (ERT): Direct and effective, especially via transdermal E2, optimal within “therapeutic window.” – Tissue-selective options: – ERβ agonists: Promote neuroprotection/anti-inflammation without ERα-mediated proliferative risks. – SERMs (e.g., Raloxifene, Bazedoxifene): Tissue-specific effects; antidepressant potential under investigation. – Phytoestrogens: Alternative pathway, but efficacy needs larger trials.

## Summary and outlook

In summary, estrogen deficiency contributes to depression through a highly integrated network of dysfunctional pathways: monoamine disruption, HPA axis hyperactivity, neurotrophic deficits, mitochondrial impairment, neuroinflammation, and gut-brain axis dysregulation. These systems do not operate in isolation but interact in a bidirectional, synergistic manner, culminating in the depressive phenotype.

Despite advances, challenges remain. A significant translational bottleneck exists, as most mechanistic evidence comes from young OVX animal models, which imperfectly mimic human perimenopause and aging. Long-term data on the efficacy and safety of ERT for depression are still scarce. Furthermore, the substantial individual variability in susceptibility and treatment response remains poorly understood. Future research must focus on bridging these gaps, identifying predictive biomarkers, and developing multi-targeted, personalized therapeutic strategies that leverage the intricate mechanisms linking estrogen to mood regulation.
